# Genomic ancestry and education level independently influence abdominal fat distributions in a Brazilian admixed population

**DOI:** 10.1371/journal.pone.0179085

**Published:** 2017-06-05

**Authors:** Giovanny Vinícius Araújo de França, Emanuella De Lucia Rolfe, Bernardo Lessa Horta, Denise Petrucci Gigante, John S. Yudkin, Ken K. Ong, Cesar Gomes Victora

**Affiliations:** 1 Post-graduate Program in Epidemiology, Federal University of Pelotas, Pelotas, Brazil, Rua Marechal Deodoro, 1160–3° Piso, Bairro Centro—Pelotas, RS; 2 Medical Research Council (MRC) Epidemiology Unit, Institute of Metabolic Science, University of Cambridge School of Clinical Medicine, Hills R, Cambridge, United Kingdom; 3 University College London, London, United Kingdom; McMaster University, CANADA

## Abstract

We aimed to identify the independent associations of genomic ancestry and education level with abdominal fat distributions in the 1982 Pelotas birth cohort study, Brazil. In 2,890 participants (1,409 men and 1,481 women), genomic ancestry was assessed using genotype data on 370,539 genome-wide variants to quantify ancestral proportions in each individual. Years of completed education was used to indicate socio-economic position. Visceral fat depth and subcutaneous abdominal fat thickness were measured by ultrasound at age 29–31y; these measures were adjusted for BMI to indicate abdominal fat distributions. Linear regression models were performed, separately by sex. Admixture was observed between European (median proportion 85.3), African (6.6), and Native American (6.3) ancestries, with a strong inverse correlation between the African and European ancestry scores (ρ = -0.93; p<0.001). Independent of education level, African ancestry was inversely associated with both visceral and subcutaneous abdominal fat distributions in men (both P = 0.001), and inversely associated with subcutaneous abdominal fat distribution in women (p = 0.009). Independent of genomic ancestry, higher education level was associated with lower visceral fat, but higher subcutaneous fat, in both men and women (all p<0.001). Our findings, from an admixed population, indicate that both genomic ancestry and education level were independently associated with abdominal fat distribution in adults. African ancestry appeared to lower abdominal fat distributions, particularly in men.

## Introduction

Obesity has become a global public health problem, reaching epidemic proportions and coexisting with under-nutrition in low and middle-income countries [1]. It is defined as a body mass index (BMI) ≥30 kg/m^2^ and constitutes an important risk factor for mortality and lifestyle-related chronic diseases, such as cardiovascular diseases, diabetes, and certain types of cancer [2]. However, as a proxy for total adiposity, BMI does not consider regional fat distribution, especially the abdominal fat depots, which have been identified as key determinants of the cardio-metabolic risks attributed to excess body weight [3].

Computed tomography and magnetic resonance imaging are reference methods that allow distinction between, and accurate quantification of the intra- and subcutaneous abdominal fat depots. Nevertheless, such methods are generally infeasible for use in large-scale epidemiological studies due to logistical, financial and ethical issues [4]. Ultrasonography has been demonstrated to be a valid, non-invasive, inexpensive, safe and widely available technique for estimating the size of abdominal fat depots [5–9]. A few previous studies evaluating intra- and subcutaneous abdominal fat by ultrasound have reported variation in these quantities according to gender, age group and ethnicity; however, those studies are generally restricted to small samples and specific age groups [5–7,10].

Several studies have described the relationships between self-reported race/ethnicity and abdominal fat using various approaches and in distinct populations [11–15]. However, self-reported race/ethnicity comprises both biological and sociocultural components [16]. Respondents may rely upon varying dimensions to categorise themselves (or others), including: ancestral descent, cultural background, and physical attributes, such as skin colour, facial features, among others [17]. It is invariably difficult to disentangle the potential effects of genetic from the socio-cultural and behavioural components of race/ethnicity on health-related outcomes, both due to their coalition in self-reports, and also due to their inter-correlation in many populations.

In Brazil, large-scale surveys typically use three skin colour categories to record self-reported race/ethnicity in the black-to-white continuum: white, brown (or ‘‘mixed”), and black [18]. In the city of Pelotas in Southern Brazil, almost 80% self-classify as white [19]. However, this population is remarkably admixed, presenting significant variation in genomic ancestry even a region that is populated predominantly by descendants of European immigrants [20]. In this setting, the use of genomic ancestry markers constitutes a far more accurate approach to quantify the admixture in each individual. Furthermore, the continuum in genomic ancestries observed in this population allows analyses to assess the independent effects of ancestry and social factors, such as education, on health-related outcomes.

In this study, we aimed to investigate the independent associations of genomic ancestry and education level with visceral and subcutaneous abdominal fat distribution assessed by ultrasound in adults participating in the 1982 Pelotas birth cohort study, Brazil.

## Materials and methods

### Study population

Pelotas is a city located in the far south of Brazil, near the border with Uruguay. In 1982, the estimated urban population of Pelotas was 214,000 and the main ethnic groups comprised European and African descendants [21]. It has a particularly high proportion of the latter, due to forced migration of slaves from West-Africa in order to work in the salted beef (jerky) industry during the 1800s [22]. The 1982 birth cohort study began as a perinatal survey and recruited 99.2% of all births occurring in Pelotas that year. The 5,914 live born infants, whose mothers lived in the urban area of Pelotas and gave birth in one of the three maternity hospitals in the city, were enrolled and constitute the original cohort.

The cohort members have been prospectively followed up on several occasions. Methodological details of the cohort and follow-up visits have been described elsewhere [21]. In the early phases, verbal informed consent was obtained from the mothers, whereas in recent phases, written consent was obtained. The latest phase of the 1982 Pelotas birth cohort study was approved by the Federal University of Pelotas Ethical Committee, which is affiliated with the Brazilian Federal Medical Council. The research has been conducted according to the principles expressed in the Declaration of Helsinki.

In 2012–13, all the members of the cohort were sought, and 73.1% (n = 4,321) were located. We examined 3,711 of them between June 2012 and February 2013. Including those members known to have died (n = 325), this sample comprises 68% of the original cohort. Valid data on visceral and subcutaneous abdominal fat thicknesses were collected by ultrasound from 3,493 participants (1,724 men and 1,769 women) aged 29–31 y. Exclusion criteria for performing abdominal ultrasound included women with a known or probable pregnancy, and those who had given birth in the previous three months. Of the 3,493, we had data on genomic ancestry for 2,890 participants (1,409 men and 1,481 women), which comprises the sample included in this analysis. This subsample did not differ from the sample of cohort members located in 2012–13 and examined using ultrasound (n = 3,493) regarding sex, education level and BMI classification (**Table 1**).

**Table 1:** Comparison between the Pelotas (Brazil) birth cohort sample with information on ultrasound measurements of abdominal fat in 2012–13 and the subsample with genomic ancestry data according to sex, socioeconomic position indicators and BMI.

Variables	Whole sample (N = 3,493)		Subsample (N = 2,890)		p-value *
	N	%	N	%	
Sex					
Male	1 724	49.4	1 409	48.8	0.63
Female	1 769	50.6	1 481	51.3	
Education (years)					
<8	626	18.1	532	18.6	0.75
8 to 11	1 301	37.7	1 088	38.0	
12 to 15	867	25.1	729	25.4	
16+	661	19.1	518	18.1	
BMI classification					
Underweight	67	1.9	50	1.7	0.89
Normal range	1 393	40.2	1 146	39.9	
Overweight	1 205	34.8	995	34.7	
Obese	799	23.1	680	23.7	

*Chi-square test.

### Phenotype measures

Abdominal ultrasound imaging was carried out using a 3.5-MHz convex probe interfaced to a Toshiba Xario (Toshiba Medical Systems Corp) ultrasound machine. Two different static images were obtained at the end of a quiet expiration by applying minimal pressure, ensuring no displacement of the abdominal cavity. For both images the probe was placed at the crossing point between the xyphoid line and the waist circumference using electronic callipers. The distance between the peritoneum boundary and the lumbar spine was considered as a proxy for the amount of intra-abdominal adipose tissue, referred herein as visceral fat thickness [23]. Total subcutaneous abdominal fat depth was measured from the posterior line of dermis to the outer bowel wall [24].

Three trained technicians using a standardized protocol performed the ultrasound scans. The technicians performed all measurements immediately after obtaining each image. Three quality control sessions were performed, each with groups of ten volunteers, by comparing the results of all three technicians to those of one investigator (GVAF) who had been previously trained and certified in this technique. The relative intra-observer technical error of measurement for the visceral thickness was 4.1% and 3.4% for subcutaneous abdominal fat thickness, and the relative inter-observer technical error of measurement was 3.1% for both measurements.

Trained research assistants performed anthropometric measurements using standard procedures. Participants were barefoot and wearing light clothing. Weight was measured to the nearest 0.1 kg on a calibrated electronic scale (TANITA model BC-418 MA; Tanita, Tokyo, Japan). Standing height was assessed to the nearest 0.1 cm using a full-length wall-mounted stadiometer (SECA 240; Seca, Birmingham, United Kingdom). Body mass index (BMI; in kg/m^2^) was calculated by dividing weight by height squared.

### Genotype measures and SEP

The genomic ancestry analysis was based on peripheral blood DNA samples of 3,736 cohort members who had been evaluated at 22–23 years of age. The analyses were carried out as part of the Epigen Initiative, as reported by Lima-Costa et al. [25]. Genotyping was performed using the Illumina Omni 2.5M array (San Diego, California). Quality control analysis of the data was performed using Genome Studio (Illumina), PLINK, GLU (code.google.com/p/glu-genetics/), Eigenstrat, and in-house scripts. [26] Admixture analyses were based on 370,539 SNPs shared by samples from the HapMap Project, the Human Genome Diversity Project (HGDP), and the Epigen-Brazil study population. Detailed information on the external parental populations and family structure are previously reported [25]. Briefly, we used the following samples from the HapMap Project as external panels: 266 Africans (176 Yoruba in Ibadan, Nigeria [YRI] and 90 Luhya in Webuye, Kenya [LWK]), 262 Europeans (174 Utah residents with Northern and Western European ancestry [CEU] and 88 from Toscans from Italy [TSI]), 170 admixed individuals (77 Mexicans from Los Angeles, California [MEX] and 83 Afro-African from Southwest USA [ASW]), and 93 Native Americans from the HGDP (25 Pima, 22 Karitiana, 25 Maya and 21 Surui). Familial structure was assessed by estimating the kinship coefficients for each possible pair of individuals from each cohort (The 1982 Pelotas birth cohort [21], The Bambui cohort [27], and The Salvador-SCAALA project [28]) using the REAP software (Related Estimation in Admixed Populations) [29]. The contributions from African, European and Native American ancestry in each individual was estimated using the ADMIXTURE software [30].

As an indicator of SEP, we used years of completed education, estimated based on interviews using a structured questionnaire applied by trained interviewers. It was categorised into the commonly achieved local educational levels: less than 8 years; 1^st^ cycle completed (8–11 years); 2^nd^ cycle completed (12–15 years); and college degree (16 years or more).

### Statistical analyses

Statistical analyses were performed using Stata version 13 (StataCorp, College Station, Texas, USA). All analyses were stratified by sex. Independent variables were presented using absolute and relative frequencies and chi-square test was used to compare population characteristics.

Inter-correlations between the ultrasound measurements and independent variables were assessed by Spearman’s correlation. For subsequent analyses, we log-transformed visceral fat thickness and square-root transformed subcutaneous abdominal fat thickness to achieve normal distributions, and both variables were posteriorly standardized to allow direct comparisons of the regression coefficients for these outcomes.

Genomic ancestry scores showed non-parametric distributions. Therefore, quintiles for each score were calculated and, in the absence of non-linearity, were analysed as continuous variables by tests for linear trend. The European ancestry score showed strong inverse correlation with the African ancestry score (ρ = -0.93; p<0.001) and therefore was not considered separately. Because non-linear associations with visceral and subcutaneous abdominal fat thicknesses were identified by incremental F-test and component-plus-residual plots, education level was included in the multivariate models as a categorical variable and p-values from heterogeneity tests are presented.

Separate multivariate models were designed to estimate the crude and adjusted regression coefficients for the associations between the independent variables (African and Native American ancestry, and education) and the outcomes (visceral and subcutaneous fat). The adjusted models included the two genomic ancestry estimates and education level in the same model, in order to estimate their independent effects on abdominal fat.

Both the ‘crude’ and ‘adjusted’ models were performed with and without further adjustment for BMI at age 29–31 years. Models without adjustment for BMI consider ‘abdominal fat thickness’ as the outcome. Model including BMI as a covariate consider ‘abdominal fat distribution’ as the (primary) outcome.

In order to explore the identified non-linear associations with education, we tested the interaction between education and sex on the associations with both visceral and subcutaneous fat thicknesses. A 5% significance level was applied.

## Results

**Fig 1:**
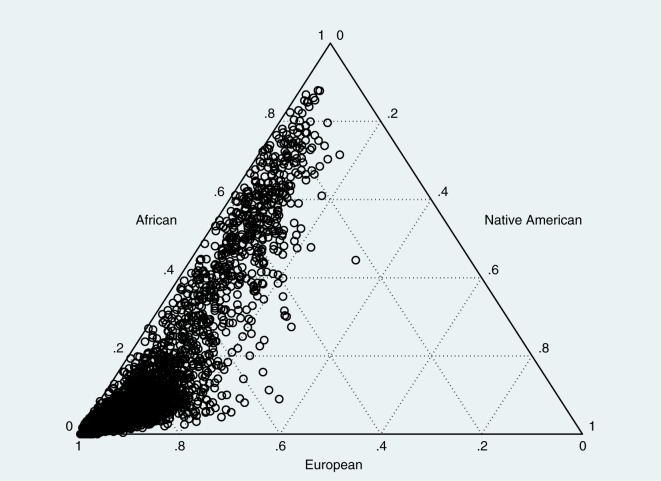
Triangle plot of the 1982 Pelotas (Brazil) birth cohort’s members according to ancestry admixture proportions. Each symbol represents an individual. Each person was genotyped and ancestry-informative markers were used to provide information on African, Native American, and European ancestry.

The study population characteristics are summarized in **Table 2**. European ancestry contributed the highest ancestry proportion in both men (median = 85.4%) and women (median = 84.9%), but with considerable inter-individual variability (**Fig 1**). Men reported less education than women (p<0.001). Both African and Native American ancestry are associated with education (p<0.001).

**Table 2:** Description of the study sample.

Variables	Men		Women		p-value
*Ancestral proportions [Median % (IQR)]*	*[n = 1 409]*		*[n = 1 481]*		
European	85.4 (73.1–91.4)		84.9 (71.3–90.7)		0.10
African	6.6 (3.7–16.5)		6.8 (3.8–17.5)		0.28
Native American	6.1 (3.6–9.6)		6.6 (4.0–9.6)		0.02
*Education level (years) [N (%)]*	*[n = 1 397]*		*[n = 1 470]*		
<8	284	(20.3)	248	(16.9)	<0.001*
8 to 11	564	(40.4)	524	(35.6)	
12 to 15	355	(25.4)	374	(25.4)	
16+	194	(13.9)	324	(22.0)	
*BMI classification [N (%)]*	*[n = 1 399]*		*[n = 1 472]*		
Underweight (<18.5 kg/m^2^)	18	(1.3)	32	(2.2)	<0.001*
Normal range (18.5–24.9 kg/m^2^)	488	(34.9)	658	(44.7)	
Overweight (25.0–29.9 kg/m^2^)	574	(41.0)	421	(28.6)	
Obese (≥30 kg/m^2^)	319	(22.8)	361	(24.5)	
*Ultrasound [Median (IQR)]*	*[n = 1 409]*		*[n = 1 481]*		
Visceral fat thickness (cm)	6.7	(2.6)	4.7	(2.0)	<0.001**
Subcutaneous abdominal fat thickness (cm)	1.8	(1.3)	2.4	(1.6)	<0.001**

*Fisher's exact test

**Two-sample Wilcoxon rank-sum (Mann-Whitney) test

Overweight was more prevalent in men than in women (p<0.001). Men had greater median visceral fat thickness than women (6.7 vs. 4.7cm; p<0.001), but lower mean subcutaneous fat thickness (1.8 vs. 2.4cm; p<0.001); these differences persisted after adjustment for BMI at 30y (all p<0.001; not shown). Visceral fat thickness showed modest positive correlations with subcutaneous abdominal fat thickness, both in men (ρ = 0.43; p<0.001) and women (ρ = 0.39; p<0.001) (**Table 3**). Additionally, both visceral and subcutaneous abdominal fat thicknesses showed strong positive correlations with BMI (all p<0.001).

**Table 3:** Correlations (Spearman’s ρ) between abdominal fat thicknesses and independent variables in men (n = 1,371) and women (n = 1,434).

Variables	Men		Women	
	ρ	p-value	ρ	p-value
**Visceral fat thickness (cm)**				
African ancestral proportion	-0.04	0.12	0.11	<0.001
Native American ancestral proportion	0	0.97	0.08	0.06
European ancestral proportion	0.04	0.15	-0.12	<0.001
Education (years)	-0.07	0.01	-0.23	<0.001
BMI (kg/m^2^)	0.67	<0.001	0.59	<0.001
**Subcutaneous abdominal fat thickness (cm)**				
African ancestral proportion	-0.11	<0.001	0.02	0.43
Native American ancestral proportion	-0.05	0.003	0.06	0.02
European ancestral proportion	0.13	<0.001	-0.05	0.08
Education (years)	0.15	<0.001	-0.03	0.19
BMI (kg/m^2^)	0.75	<0.001	0.82	<0.001

Note: Correlations between visceral and subcutaneous fat thicknesses: men (ρ = 0.43; p<0.001) and women (ρ = 0.39; p<0.001).

### Associations with abdominal fat thickness

In men, the crude inverse association (p = 0.001) between African ancestry and subcutaneous fat thickness was attenuated after adjustment for education (p = 0.05) (**Table 4**). In women, the crude positive associations between African ancestry (p<0.001) or Native American ancestry (p = 0.01) and visceral fat thickness were also attenuated after adjustment for education (p = 0.26 and p = 0.59, respectively).

**Table 4:** Crude and adjusted regression coefficients (standardized)^1^ for visceral and subcutaneous abdominal fat thickness according to ancestry markers and socioeconomic position indicators.

Variables	Visceral fat thickness^2^						Subcutaneous abdominal fat thickness^3^					
	*Crude*			*Model 1* ^*4*^			*Crude*			*Model 1* ^*4*^		
	β	S.E.	p-value	β	S.E.	p-value	β	S.E.	p-value	β	S.E.	p-value
**Men**				*R*^*2*^ *= 0*.*01 *						*R*^*2*^ *= 0*.*03*		
***Ancestry markers***												
African^5^	-0.02	0.01	0.11	-0.04	0.02	0.03	-0.06	0.02	0.001	-0.04	0.02	0.05
Native American^5^	0.00	0.01	0.82	0.01	0.02	0.46	-0.03	0.02	0.10	0.00	0.02	0.79
***Education (years)***												
<8	Ref		0.01	Ref		0.003	Ref		<0.001	Ref		<0.001
8 to 11	-0.11	0.05		-0.17	0.06		0.27	0.06		0.25	0.07	
12 to 15	-0.10	0.06		-0.14	0.06		0.42	0.07		0.44	0.07	
16 or more	-0.22	0.07		-0.27	0.08		0.43	0.08		0.39	0.09	
**Women**	** **	** **	** **	*R*^*2*^ *= 0*.*05*			** **	** **	** **	*R*^*2*^ *= 0*.*01*		
***Ancestry markers***												
African^5^	0.07	0.02	<0.001	0.02	0.02	0.26	0.01	0.02	0.43	0.00	0.02	0.82
Native American^5^	0.05	0.02	0.01	0.01	0.02	0.59	0.04	0.02	0.05	0.03	0.02	0.11
***Education (years)***												
<8	Ref		<0.001	Ref		<0.001	Ref		<0.001	Ref		0.02
8 to 11	-0.11	0.06		-0.09	0.07		0.16	0.07		0.15	0.08	
12 to 15	-0.36	0.07		-0.37	0.07		0.20	0.07		0.18	0.08	
16 or more	-0.59	0.07		-0.52	0.08		-0.05	0.07		0.00	0.09	

^1^ β approximates the SD change in outcome per 1-SD change in the exposure.

^2^ Standardized means of log-transformed visceral fat thickness (cm).

^3^ Standardized means of square-root transformed total subcutaneous abdominal fat thicknesses (cm).

^4^ Model 1: Both ancestry variables and education level adjusted in the same model.

^5^ As quintiles, included in the model as continuous.

Independent of genomic ancestry, education level was inversely associated with visceral fat thickness, both in men and women (both p<0.001). In contrast, we found non-linear associations between education level and subcutaneous abdominal fat thickness, both in men and women. In women, there was an inverted U-shaped relationship between education level and subcutaneous abdominal fat thickness.

**Fig 2:**
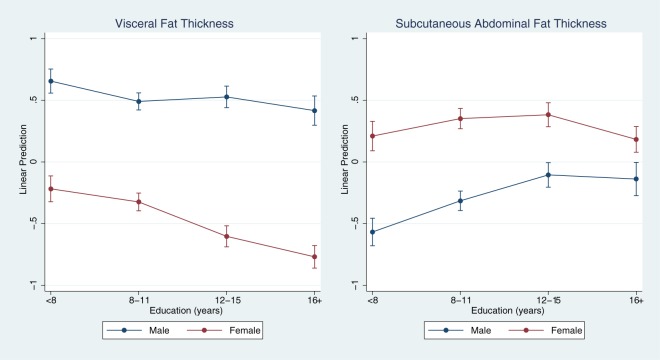
Adjusted means of visceral and subcutaneous abdominal fat thicknesses according to completed years of education and sex. Both ancestry variables and education level adjusted for each other. Both p-interaction < 0.001.

Sex significantly modified the associations of education with both visceral and subcutaneous abdominal fat thicknesses (both p-interaction<0.001) (Fig 2). In women, lower education was associated with greater visceral fat thickness, but lower subcutaneous abdominal fat thickness.

### Associations with abdominal fat distribution

In men, African ancestry was inversely associated with both visceral and subcutaneous abdominal fat distributions, after adjustment for education level (both p<0.001) (**Table 5**). Similarly, in women, African ancestry was inversely associated with subcutaneous abdominal fat distribution (p = 0.009), but not with visceral fat distribution. Native American ancestry score was not associated with either visceral or subcutaneous abdominal fat distribution in men or women.

**Table 5:** Standardized regression coefficients^1^ for visceral and subcutaneous abdominal fat distribution according to ancestry markers and socioeconomic position indicators, adjusted for current BMI.

Variables	Visceral fat distribution^2^						Subcutaneous abdominal fat distribution^3^					
	*Bivariate models*^*4*^			*Multivariate model*^*5*^			*Bivariate models*^*4*^			*Multivariate model*^*5*^		
	β	S.E.	p-value	β	S.E.	p-value	β	S.E.	p-value	β	S.E.	p-value
***Men***				*R*^*2*^ *= 0*.*46**** ***						*R*^*2*^ *= 0*.*57**** ***		
***Ancestry markers***												
African^6^	-0.02	0.01	0.09	-0.04	0.01	0.001	-0.05	0.01	<0.001	-0.04	0.01	0.001
Native American^6^	0.02	0.01	0.17	0.02	0.01	0.06	-0.01	0.01	0.24	0.01	0.01	0.47
***Education (years)***												
<8	Ref		<0.001	Ref		<0.001	Ref		<0.001	Ref		<0.001
8 to 11	-0.15	0.04		-0.19	0.04		0.22	0.04		0.22	0.05	
12 to 15	-0.18	0.04		-0.21	0.05		0.34	0.05		0.36	0.05	
16 or more	-0.26	0.05		-0.30	0.06		0.37	0.05		0.35	0.06	
***Women***				*R*^*2*^ *= 0*.*40*** **						*R*^*2*^ *= 0*.*63*** **		
***Ancestry markers***												
African^6^	0.03	0.01	0.02	0.00	0.01	0.91	-0.03	0.01	0.002	-0.03	0.01	0.009
Native American^6^	0.02	0.01	0.08	0.00	0.01	0.98	0.00	0.01	0.84	0.02	0.01	0.15
***Education (years)***												
<8	Ref		<0.001	Ref		<0.001	Ref		<0.001	Ref		<0.001
8 to 11	-0.15	0.05		-0.14	0.05		0.12	0.04		0.10	0.05	
12 to 15	-0.32	0.05		-0.32	0.06		0.28	0.05		0.28	0.05	
16 or more	-0.45	0.06		-0.43	0.06		0.18	0.05		0.14	0.05	

^1^ β approximates the SD change in outcome per 1-SD change in the exposure.

^2^ Standardized means of log-transformed visceral fat thickness (cm).

^3^ Standardized means of square-root transformed total subcutaneous abdominal fat thicknesses (cm).

^4^ Bivariate models: One model for each variable, all adjusted for BMI.

^5^ Multivariate models: Both ancestry variables and education level adjusted for each other and for BMI.

^6^ As quintiles, included in the model as continuous.

Independent of genomic ancestry, education level was inversely associated with visceral fat distribution, both in men and women (both p<0.001). In contrast, education level showed positive but non-linear associations with subcutaneous abdominal fat distribution, both in men and women (both p<0.001).

## Discussion

To our knowledge, this is the first study to apply genome-wide markers to investigate the effects of ancestry on abdominal fat distribution. It is also one of the largest reported samples with abdominal ultrasound measurements, and by far the largest from a low- or middle-income setting. Our findings, from an admixed population, indicate that both genomic ancestry and education level were independently associated with abdominal fat distribution in adults. African ancestry was inversely associated with visceral and subcutaneous abdominal fat in men, and was inversely associated with subcutaneous abdominal fat in women. Regarding abdominal fat thickness (i.e. unadjusted for BMI), apparent associations with ancestry were likely due to confounding by lower educational level in individuals with higher proportions of African or Native American ancestry.

Ancestry markers have been used to explore the aetiology of complex diseases. Previous studies using specific panels of genomic ancestry markers have reported associations with obesity-related phenotypes [31–33]. Goonesekera et al. [32], analysing a sample of 1,726 individuals participating in the Boston Area Community Health survey, a population-based prospective cohort study, observed positive associations of West-African ancestry with BMI and percent body fat. Moreover, gender significantly modified the association between West-African ancestry and BMI by gender (p-interaction: <0.002), with greater magnitude among women. Klimentidis et al. [33], studying 2814 self-identified African-American (AA) participating in the Atherosclerosis Risk in Communities study and 1611 AA from the Multi-Ethnic Study of Atherosclerosis, found a negative association between West-African ancestry and central obesity among African-American men, but not among women (pinteraction = 4.14 × 10−5 in pooled analysis of WHR). Consistent with our findings, a previous study reported a positive association between European ancestry and visceral fat in an African American sample [34].

While such studies using genomic ancestry markers are yet sparse, these findings are consistent with some earlier studies that used self-identified race/ethnicity, which reported that African Americans have lower visceral fat than white Americans, independent of total body fat [12,35,36]. Although the specific genetic factors behind these racial differences are yet to be identified, our study strongly supports the existence of biological effects of genomic ancestry on the accumulation and distribution of abdominal fat depots.

The relationship between SEP and abdominal fat distribution is complex in nutritionally transitioning cohorts in middle-income countries, such as Brazil. Sobal and Stunkard’s [37] landmark review in 1989 described an inverse association between SEP and obesity among women in high-income countries. Since then, such inverse associations have also emerged in middle-income countries and represent a reversal from the positive associations that are still seen in low-income countries, especially among men [38,39]. Regarding abdominal adiposity, a previous analysis of the 1982 Pelotas (Brazil) birth cohort showed positive associations for family income in adulthood with waist and hip circumferences, both partially mediated by education and behavioral variables [40].

In the current study, effect estimates on abdominal fat for education level were substantially larger than those with ancestry; higher education level was robustly associated with lower visceral fat thickness and distribution, but conversely with higher subcutaneous abdominal fat distribution. In addition, we found that women with lower education tend to present greater visceral fat thickness and lower subcutaneous abdominal fat thickness in comparison with those with higher education, suggesting a more detrimental profile in terms of metabolic risk.

The reason for these apparent directionally-discordant effects on visceral versus subcutaneous abdominal fat is unclear, but might reflect age- and cohort-specific contributions of SEP. In adults, higher education has been also suggested to be associated with expectations for personal achievement in several aspects, affecting personal satisfaction with body image and perception of desirable body shape, especially among women [41].

We acknowledge some limitations of our study. Firstly, we used ultrasound measurements of abdominal visceral and subcutaneous fat thickness, as proxies for these abdominal fat masses. While the validity of ultrasound in this specific setting has not been examined, validation studies using the same standardized protocol have found strong correlations between ultrasound and MRI estimates of abdominal fat in a variety of settings and populations [6,7,23]. In addition, our findings are consistent with previous reports using different imaging methods that men have greater intra-abdominal and lower abdominal subcutaneous adipose tissue than women [42,43]. Secondly, the Native American ancestry proportion was low in our sample, as it is in South Brazil, and similar studies in other settings are needed to better examine its potential influence on markers of health and disease risk. Consequently, there was high correlation between the remaining two ancestries, and our findings related to African ancestry should be interpreted relative to European ancestry.

In conclusion, use of genomic ancestry in this uniquely admixed population allowed inference of independent biological and social influences on abdominal fat distribution. African ancestry appeared to lower abdominal fat distributions, particularly in men.
