# Correction: Genomic ancestry and education level independently influence abdominal fat distributions in a Brazilian admixed population

**DOI:** 10.1371/journal.pone.0196265

**Published:** 2018-04-17

**Authors:** The PLOS ONE Staff

The following information is missing from the Data Availability section: The dataset is available at the following link: https://figshare.com/articles/PONE-D-17-02552R/5971333.

The publisher apologizes for the error.
